# A qualitative exploration of the challenges providers experience during peripartum management of patients with a body mass index ≥ 50 kg/m^2^ and recommendations for improvement

**DOI:** 10.1371/journal.pone.0303497

**Published:** 2024-05-16

**Authors:** Michelle A. Kominiarek, Madison Lyleroehr, Jissell Torres

**Affiliations:** 1 Division of Maternal-Fetal Medicine, Department of Obstetrics and Gynecology, Northwestern University Feinberg School of Medicine, Chicago, IL, United States of America; 2 Department of Medical Social Sciences, Northwestern University Feinberg School of Medicine, Chicago, IL, United States of America; Deakin University Faculty of Health, AUSTRALIA

## Abstract

**Background:**

The objective of this research was to conduct a qualitative study among a diverse group of providers to identify their clinical needs, barriers, and adverse safety events in the peripartum care of people with a body mass index (BMI) ≥ 50 kg/m^2^.

**Methods:**

Obstetricians, anesthesiologists, certified nurse midwives, nurse practitioners, and nurses were invited to participate in focus group discussions if they were employed at the hospital for >6 months. Key concepts in the focus group guide included: (1) Discussion of challenging situations, (2) Current peripartum management approaches, (3) Patient and family knowledge and counseling, (4) Design and implementation of a guideline (e.g., checklist or toolkit) for peripartum care. The audiotaped focus groups were transcribed verbatim, uploaded to a qualitative analysis software program, and analyzed using inductive and constant comparative approaches. Emerging themes were summarized along with representative quotes.

**Results:**

Five focus groups of 27 providers were completed in 2023. The themes included staffing (level of experience, nursing-patient ratios, safety concerns), equipment (limitations of transfer mats, need for larger sizes, location for blood pressure cuff, patient embarrassment), titrating oxytocin (lack of guidelines, range of uses), monitoring fetal heart rate and contractions, patient positioning, and communication (lack of patient feedback, need for bias training, need for interdisciplinary relationships). Providers gave examples of items to include in a “BMI cart” and suggestions for a guideline including designated rooms for patients with a BMI ≥ 50 kg/m^2^, defining nursing ratios and oxytocin titration plans, postpartum incentive spirometer, and touch points with providers (nursing, physicians) at every shift change.

**Conclusions:**

Providers discussed a range of challenges and described how current approaches to care may negatively affect the peripartum experience and pose threats to safety for patients with a BMI ≥ 50 kg/m^2^ and their providers. We gathered information on improving equipment and communication among providers.

## Introduction

In 2015–2016, 41.1% of women who were 20 years of age or older in the United States had obesity, defined as a body mass index (BMI) ≥ 30 kg/m^2^ [1]. These statistics translate into a significant proportion of people at risk for having obesity during pregnancy. Obesity is an increasingly common challenge in contemporary labor management [2]. People with obesity are more likely to have an induction of labor and experience labor abnormalities [2]. Evidence also suggests a positive correlation between weight and the amount of intravenous oxytocin needed to deliver vaginally in either induced or augmented labor [3–8]. As such, titration of oxytocin during labor can be challenging if high infusion rates have been reached without cervical change. Obesity also increases the risk for cesarean delivery and its related morbidities such as surgical site infection, postpartum hemorrhage, and thromboembolism [9]. These risks are further amplified in people with class III obesity (BMI ≥ 40 kg/m^2^) [10].

Morbidity and mortality related to pregnancy is a key priority area in patient care. There is limited evidence to guide care for people in the highest weight category or a BMI ≥ 50 kg/m^2^. Given the prevalence of obesity and obesity-related complications, it is important to identify opportunities to provide safe care immediately before, during, and after delivery, or peripartum care.

Most studies on provider experiences in the management of obesity have focused on prenatal care and relate to topics such as gestational weight gain [11, 12]. These provider experiences have primarily been from providers such as nurse midwives or nurse practitioners and lack a multidisciplinary focus [12, 13]. Furthermore, the prior studies do not address the management of the extremes of obesity. The purpose of this research was to conduct a qualitative study in which a diverse group of providers (e.g., obstetricians, anesthesiologists, nurse midwives, nurse practitioners, nurses) identified their clinical needs, barriers to optimal care, and safety events in the peripartum care of people with a BMI ≥ 50 kg/m^2^. Given that most adverse outcomes occur surrounding the time of delivery for people with a BMI ≥ 50 kg/m^2^, identifying and processing this information from obstetrical providers is an important next step in identifying best practices for peripartum care.

## Materials and methods

Northwestern Medicine’s Prentice Women’s Hospital is the largest delivery hospital in Illinois, with over 11,000 deliveries every year. At our site, approximately 50% of patients have a BMI ≥ 30 kg/m^2^ and 10% have a BMI ≥ 40 kg/m^2^. Thus, our providers have a depth of experience in the management of patients with higher BMIs. Obstetric safety events are considered key priority areas in patient care. Adaptations already in place at this site for patients with a BMI ≥ 50 kg/m^2^ prior to the study were placement of a transfer mat on the patient’s bed at delivery admission, making a note in the electronic medical record dashboard regarding the BMI, and using 3g instead of 2g of cefazolin for antibiotic prophylaxis prior to a cesarean delivery.

The Northwestern University IRB approved this study. Reporting for this qualitative study was done in accordance with the Consolidated criteria for reporting qualitative research (COREQ) checklist (S1 File) [14]. The principal investigator (MAK) had prior experience with both quantitative and qualitative research with pregnant persons and their providers. The potential participants had previously worked with the principal investigator in clinical and research contexts. Providers were identified from departmental list-serves at the site. Providers were invited via email (purposive sampling) to participate in a one-time focus group to assess their experiences in the care of people with a BMI ≥ 50 kg/m^2^ and elicit barriers and facilitators to optimal peripartum care. Providers were eligible if they were a physician (faculty, resident, fellow) in obstetrics or anesthesia, certified nurse midwife (CNM) or nurse practitioner (NP) in labor and delivery or postpartum, surgical assistant in the labor and delivery operating room, or a labor and delivery or postpartum registered nurse. Providers were also eligible if they were employed at the site for at least 6 months and were English-speaking. Focus groups were scheduled such that only people of the same provider type were in a group. The sample pool size was >100 faculty physicians, >100 nurses, >50 resident and fellow physicians, >20 certified nurse midwives or nurse practitioners, and <5 surgical assistants. Recruitment occurred between February 1, 2023 and May 24, 2023.

In person focus groups, estimated to last 45–60 minutes each, were scheduled in a hospital conference room when 3–5 providers indicated an availability to participate. Each provider was given a $50 gift card and a meal as compensation for their participation. The goal was to complete 4–5 focus groups with a total of 25–30 participants and 4–6 participants in each group. To help reach participation targets, individual interviews were presented as an option for providers who were interested in participating in the study but were unable to attend their group’s scheduled session. All focus groups were led by a master’s level (MA) female team member (ML), a research analyst with a decade of experience in qualitative research who did not know the participants prior to the study and vice versa. No specific characteristics were reported to the participants regarding the interviewer. The principal investigator (MAK) was also present during most of the focus groups, as was a research coordinator (JT), who took notes when present. Once informed written consent was obtained, participants then completed a 16-item questionnaire (“Provider Characteristics and Practice Patterns”) which contained closed and open-ended questions to assess practice specialty, years in practice, current approach to peripartum care of people with a BMI ≥ 50 kg/m^2^, and current use of any guidelines for peripartum care. The principal investigator designed the survey, and it was available for completion online via REDCap or on paper at the focus group session (and entered into REDCap afterwards), per the participants’ preference [15, 16]. Key concepts in the focus group guide included: (1) Discussion of challenging situations for BMI ≥ 50 kg/m^2^, (2) Current peripartum management approaches, (3) Patient and family knowledge and counseling, (4) Design and implementation of a guideline (e.g., checklist or toolkit) for peripartum care of people with a BMI ≥ 50 kg/m^2^. Providers were encouraged to share their experiences in particularly difficult cases. The same focus group guide (S2 File) was used for all data collection sessions, and no edits were made to the guide during the data collection period.

The 16-item questionnaire data were summarized, and descriptive statistics were reported for the sample (S3 File). Responses to open-ended questions were sorted, organized, and checked with focus group and interview data for any significant discrepancies. The audiotaped focus groups were transcribed verbatim by a 3^rd^ party transcription system. Transcripts from the focus groups and interview were uploaded into the qualitative analysis software program, Dedoose version 9.0.46 (Los Angeles, California) and analyzed using inductive and constant comparative approaches [17, 18]. The first three focus group transcripts were reviewed by ML using descriptive coding to create a preliminary codebook [19]. Transcripts were not returned to participants for comment or correction and participants did not provide feedback on the findings. The codebook (S4 File) included definitions and instructions for application of each code to ensure uniformity and avoid overlap during coding. The same three transcripts were then coded using the preliminary codebook. Additional details on code definitions were added as appropriate throughout the coding process. At the end of the coding process, some conceptually related codes were combined due to low usage.

Once coding was complete, reports for each individual code were downloaded from Dedoose. Every excerpt for each code was reviewed for uniformity. Each report was then summarized and reviewed for emergent themes, which were organized and summarized, along with representative quotes.

## Results

Five focus groups were completed in 2023, along with one interview for a provider who was unable to attend a scheduled focus group. Invited surgical assistants did not respond to the email invitations. Six additional providers (n = 2 nurses, n = 1 anesthesiology physician, n = 3 resident physician) were scheduled to attend a focus group but were not able to attend. In total, 27 providers participated. The demographics of the participants, as well as information about their clinical role and training, are detailed in Table 1. Most participants were non-Hispanic White females, per self-report. Some providers’ (obstetrics-gynecology residents, anesthesiology fellows) time at the site was < 1 year, whereas some of the other providers reported longer employment at the site (40 years). Most participants made some type of adaptation to either intrapartum (92.6%) or postpartum (74.1%) care of patients with a BMI ≥ 50 kg/m^2^.

**Table 1:** Participant demographic characteristics.

	n = 27 n (%)
**Age in years**	
Mean (range)	38.4 (24–63)
**Gender**	
Male	4 (14.8%)
Female	22 (81.5%)
Missing	1 (3.7%)
**Race**	
White	22 (81.5%)
Black	2 (7.4%)
Missing/other	3 (11.1%)
**Ethnicity**	
Hispanic	3 (11.1%)
Non-Hispanic	22 (81.5%)
Prefer not to answer	2 (7.4%)
**Job/Role **	
Faculty–OB/GYN	4 (14.8%)
Faculty–Obstetrical Anesthesiologist	2 (7.4%)
Fellow–Obstetrical Anesthesiologist	4 (14.8%)
Resident–OB/GYN	4 (14.8%)
CNM or NP	7 (25.9%)
RN	6 (22.2%)
**Group **	
Faculty OB/GYN	4 (14.8%)
Anesthesiologist (faculty and fellows)	6 (22.2%)
Resident OB/GYN	4 (14.8%)
CNM or NP	7 (25.9%)
RN	6 (22.2%)
**Number of years at site**	
Median (interquartile range)	6 (2.5–16)
**Number of years since completing training **	
Median (interquartile range)	4 (2–17)
**Do you make any modifications for the intrapartum care of people with a BMI ≥ 50 kg/m** ^**2**^ **? **	
Yes	25 (92.6%)
No	1 (3.7%)
Missing	1 (3.7%)
**Do you make any modifications for the postpartum care of people with a BMI ≥ 50 kg/m** ^**2**^ **? **	
Yes	20 (74.1%)
No	7 (25.9%)
**Are there any specific guidelines that you follow for the peripartum care of people with a BMI ≥ 50 kg/m** ^**2**^ **? **	
Yes	14 (51.9%) *
No	10 (37%)
Not applicable	3 (11.1%)

*Examples of specific guidelines included the American College of Obstetricians and Gynecologists (n = 1 provider) and Society for Obstetric Anesthesia and Perinatology (n = 1 provider)

BMI body mass index

OB/GYN obstetrician-gynecologist

CNM certified nurse practitioner

NP nurse practitioner

RN registered nurse

Providers discussed a range of challenges, concerns, and needs regarding care for patients with a BMI ≥ 50 kg/m^2^. They discussed how aspects of care may negatively affect the labor and birth experience for these patients. Findings are presented by topic, so the barriers specific to each topic are discussed alongside implications for patients and opportunities for improvement.

**Fig 1:**
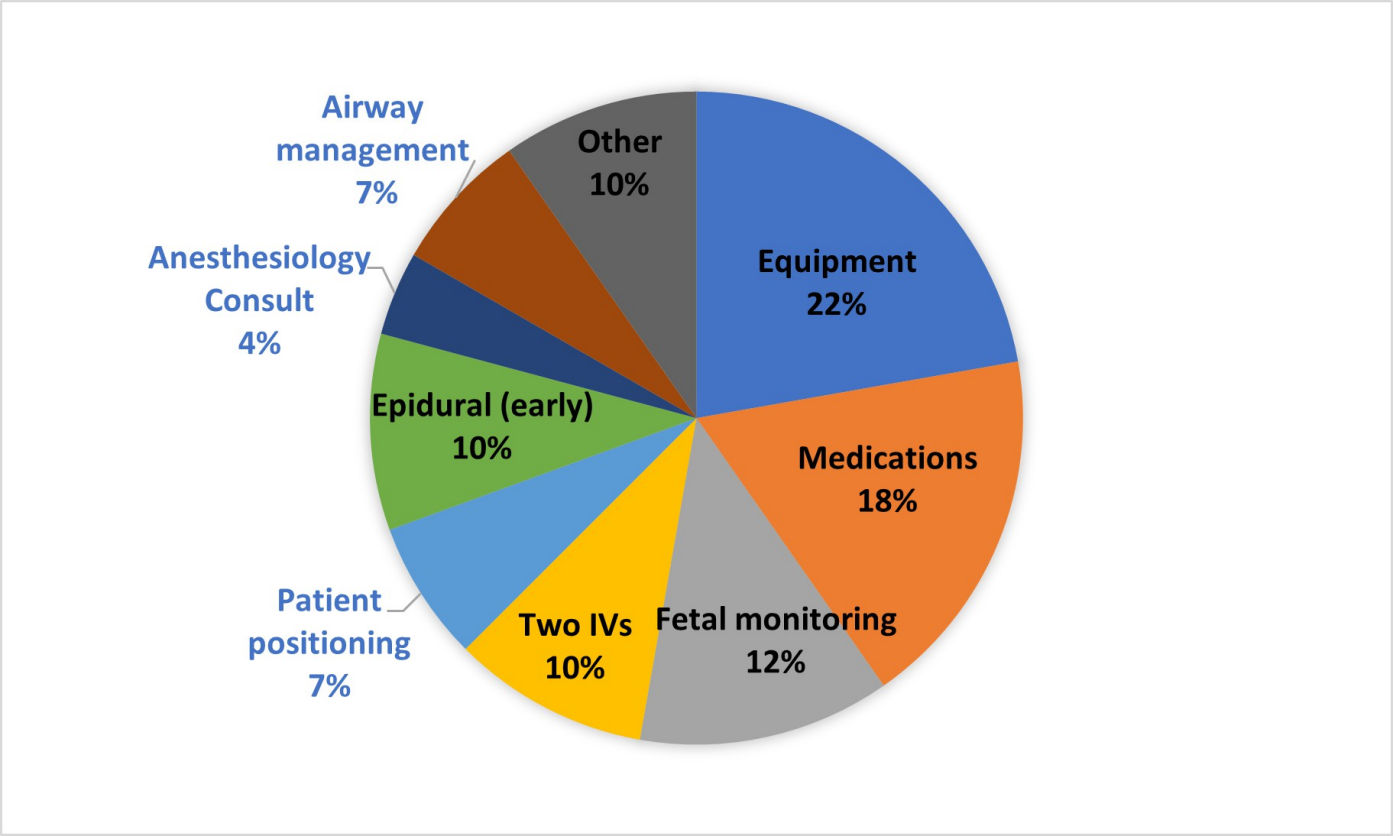
Provider modifications for patients with a BMI ≥ 50 kg/m^*2*^ based on responses to 16-item questionnaire.

The survey asked open-ended questions about what care modifications providers currently make for patients with a BMI ≥ 50 kg/m^2^. This distribution shows that many aspects of care are affected for patients with a BMI ≥ 50 kg/m^2^, which is consistent with the live discussions. (Fig 1)

### Staffing

While staffing was only mentioned once as a modification for patients with a BMI ≥ 50 kg/m^2^ (represented in the “Misc.” category in Fig 1) in the open-ended questions on the survey, it was one of the major concerns the providers discussed during the focus groups. They reported needing more confidence and experience in major areas of care, like moving patients, monitoring contractions and fetal heart tones, and performing fundal checks. Providers found many tasks more difficult, such as constantly adjusting the monitors, trying to position patients, and keeping transfer mats in place. Additional staff help was often needed. Level of nursing experience closely tied into their actions in the labor course, such as this story from a nurse:

I think when I started as a nurse, I had a really hard time tracing higher BMI patients. But I was tracing this patient super, super well for all 12 hours of my shift. Come night shift, a newer nurse came on, did not trace the patient super well. And within 30 minutes, they had put internal monitors in that patient and didn’t even give it a second thought. So, now that patient is at increased risk of chorio. I think when things like that happen, it’s a little bit frustration.

Providers reported that providing care for patients with a BMI ≥ 50 kg/m^2^ requires more clinical judgement and effort, which increases the difficulty in making labor and delivery decisions. One obstetrics-gynecology (OBGYN) faculty explained:

Decision to deliver can be very stressful, and if you are in a situation where you’re trying to deliver rapidly, the logistics of getting the patient transferred to the operating room, confirming there’s a level, and then just deciding about how to optimize your access–where to make the incision–all takes more time and thoughtfulness than in a non-super-obese patient. And so, I think the time delay there, you always think about that, and that’s something that’s unique in the care of that population. I think that sometimes influences your willingness to continue along the labor course, or to proceed more towards that delivery.

Finally, providers lamented that the increased time, effort, and additional help negatively affects other patients receiving care at the same time. Because providers spent more time with patients with a BMI ≥ 50 kg/m^2^, they had less time to spend with their other patients, affecting the patient experience for all.

The staffing challenges providers described contributed to two primary safety concerns that came up during discussions. First, providers discussed how performing some responsibilities, especially positioning and moving patients, can be a safety issue for patients as well as providers, especially when attempting to move patients on their own. One OB/GYN resident described this concern in detail:

And I think that maybe we should have–should we have like two to one nursing for some patients who have a higher BMI, or just have help for being able to help move them into different positions, like left, right, getting their legs over the peanut ball, sometimes. And also, it can sometimes be like a workforce injury or something, to smaller nurses who are trying to move very big patients; it’s also not safe for the nurses, sometimes. And so, having two people, or other mechanisms to help move patients, would be safer for our staff, and safer for them, ultimately, if that has any impact on their labor course, and decreasing C sections, and things like that.

Second, providers also expressed concerns regarding not being able to identify certain potentially serious issues. For example, a nurse described challenges around doing fundal checks:

You see these patients sometimes go their whole recovery and things seem fine. And then, they move right before they move to postpartum and they’re hemorrhaging because the whole time, it was so difficult to really feel the top of the fundus. That just makes it more difficult to get a really reliable check. And I think these patients have just such a disservice done to them because then, they end up in these situations that might not have been avoided because sometimes, if you’re going to bleed, you’re going to bleed. But it could have been caught earlier or prevented.

To address the various staffing barriers, both nurses and physicians supported changing to a two to one or one to one staffing model, either at key points during labor or for the entire labor course. They argued the advantages of this model could be better positioning, safer labor, better patient experience, and increased likelihood of a vaginal birth. A nurse described her thoughts on this type of staffing model:

When you’re able to be singled with a BMI patient, your care is so much better. You’re not worried about watching another baby on the monitor. You can actually be in there adjusting monitors as needed, flipping them as needed, and giving them the time because it takes extra time as we’ve all alluded adjusting the monitors and adjusting on the meds and everything. But just being able to give them the time. That way, it’s just so much better. And they have a better labor because you’re able to give them that experience.

Other suggestions for improving staffing included getting more help to move patients, designating a staff member to stock BMI supplies as well as having a designated BMI team. As one nurse stated, “*People should know that when they’re going in there and when someone is making assignments to know this girl has a BMI patient*. *Let’s not give her twins also in the other room*.”

### Equipment

In terms of equipment, providers primarily criticized the transfer mats. While they acknowledged the mats were helpful, they also caused various frustrations for providers trying to optimize labor. Per the providers’ experiences, the mats often slid low in the bed and got easily bunched under patients because there was no way to secure them to the bed. Providers sometimes tried to secure them by tying them to the bed or placing them under a fitted sheet. However, these solutions can create additional challenges. As one nurse explained, “*in an emergency*, *if nobody has scissors on them*, *you can’t get the transfer mat off of the bed to transfer the patient to the OR* [operating room] *table*. *So*, *that ended up being kind of a barrier because it eliminated one problem and caused another*.” Another nurse highlighted the effect the transfer mat can have on other aspects of care, adding:

So, what ends up happening is if you are actually turning your patient every hour or every half hour or whatever they need to be doing, the [transfer] mat gets all bunched and crazy underneath the patient. And especially when you’re flipping your patient hourly, it’s one nurse in there. And you can’t be adjusting the [transfer] mat and flipping the patient at the same time. It physically can’t happen. And so, I do think that our patients end up suffering because they don’t move as much and then, they don’t dilate as quickly. And then, they get infections. And it just kind of all snowballs.

Other equipment issues largely revolved around insufficient sizing. For example, providers mentioned the hospital gowns weren’t the correct size, the fetal monitoring belts were too thin, and phalanges on breast pumps were too small for their patients. In addition, they had troubles finding the correct IV needle and blood pressure cuff sizes. Finally, providers had issues keeping the proper equipment stocked in a streamlined manner.

Providers reported that using incorrect sized equipment led to safety issues. Also, when providers used the incorrect size blood pressure cuffs, they tended to place them on locations besides the upper arm. Providers worried that readings were inaccurate and inconsistent, creating additional issues in care. A resident described the various issues involving the blood pressure cuffs:

Another thing that I think about with our patients with a higher BMI is whenever I go into their rooms, it’s so variable where their blood pressure cuff will be on their body, and I get concerned about the accuracy of those readings, and if we are detecting accurate pressures for them, especially because they oftentimes will have more complex comorbidities. And so, not being aware of if they’re ever having an evolving preeclampsia picture is something I worry about. So, I think it might be helpful for us to know, like where is their blood pressure cuff currently, with the readings that we’re seeing? Is it on their wrist? Is it actually on their forearm? And I think we have different blood pressure cuff sizes, but very frequently patients always say that it’s super uncomfortable and they can’t tolerate it, so they get moved.

This resident, along with other providers, worried that incorrect sizes of bands, gowns, and blood pressure cuffs were uncomfortable and caused unnecessary embarrassment, therefore negatively impacting patients’ birth experience. In summary, the primary equipment needs were to have a stock of larger, wider range of sizes for various labor-related equipment so that patients can be comfortable, and measurements can be accurate.

When discussing safety concerns around patient airways, the CNM/NP group wondered why incentive spirometers were not widely utilized. One participant in the group summarized this need:

The other thing that I get called on when I’m covering the post-partum pager is O2 sats that drop in the post-partum period. I feel like I get quite a bit of calls about that. And I think for any patient after abdominal surgery, there is that concern for atelectasis after surgery. For some of these larger BMI patients who might also have like underlying sleep apnea, that just puts them at risk for all kinds of things post-partum. I really wish that we had more of an emphasis on utilizing incentive spirometry on post-partum with some of these patients who are at higher risk, too.

This group wished to see incentive spirometers used post-partum to aid with airway issues more commonly experienced by patients with a BMI ≥ 50 kg/m^2^. They mentioned how this equipment was used in other areas of the hospital, but they did not see it often where they worked. To implement use, the incentive spirometer would need to be ordered and the nurses would need to be taught how to use them in order to instruct patients.

Discussion across provider groups stressed the need for creating designated rooms, areas, or trays that are consistently stocked with the equipment most often used. Table 2 below and the certified nurse midwife and nurse practitioner group summarize this need:

CNM/NP 2: But maybe that’s why it would be better if it was a designated room. Then, you know the stocking, when they come in and stock the rooms, they’re also stocking the BMI cart. So, as opposed to relying on the non-traditional stockers as an added task.

CNM/NP 1: The same way we check off the code carts. So, there is a designated person who checks off code carts every shift. Implementing that for the high-risk rooms because morbid obesity or high BMI is included in high risk.

**Table 2:** Suggested items for a BMI cart or BMI designated room.

Items
Large blood pressure cuffs
Large gowns, including one for partner (s)
Transfer mats
Large speculums
Larger IV sizes
Longer fetal monitoring belts
Larger abdominal binders (for postpartum)

BMI body mass index

While transfer mats were the most discussed piece of equipment, providers gave no suggestions on how to improve their use or keep them from sliding.

### Titrating oxytocin

Providers felt as though guidance on titrating oxytocin to create adequate contractions for patients with a BMI ≥ 50 kg/m^2^ was lacking. Many sensed that patients with a BMI ≥ 50 kg/m^2^ required more oxytocin, but providers varied in how they approached the amount and timing of such increases in oxytocin. Several providers, including nurses themselves, mentioned that nurses are hesitant to adjust oxytocin levels above what is recommended for the general population. Communication on how to proceed differed depending on who they asked, adding to confusion.

Providers desired information on how to approach oxytocin, including the pharmacokinetics of oxytocin. Some suggested that talks (even if just on a recorded video) from physicians providing guidance and scientific justification on why it is appropriate to increase oxytocin levels would be helpful. They also wanted to see standards and streamlined communication regarding titration for patients over a certain BMI, so the approach is not so provider specific, as discussed below by one of the CNM/NP group participants:

Communication with the floor nursing staff in terms of Pitocin titration. We know that patients need more Pitocin when they have a higher BMI, but we struggle a little bit with that coordination. A lot of times, the patients–we can’t pick up contractions or the nurses are a little uncomfortable going above 20 or going above 30. So, maybe just, again, communication with everyone of yes, we know that these patients are going to require more Pitocin.

### Monitoring

Providers across groups described major difficulties in monitoring contractions and fetal heart tones throughout the labor course. Even once established, keeping up monitoring was challenging, especially while moving or repositioning patients. Providers perceived that patients sometimes feel at fault when monitoring is lost since patients apologize and try not to move too much, yet providers know that staying still during labor is uncomfortable, difficult, and not ideal for labor progression. Monitoring belts often didn’t fit patients with a BMI ≥ 50 kg/m^2^.

Because of monitoring challenges, providers tended to transition to internal monitoring earlier than usual. These issues also compounded other safety concerns since monitoring is how providers measure contractions, determine the effectiveness of oxytocin, and make delivery decisions. One resident summed up many of these challenges:

We just have a lot of difficulties dealing with fetal heart tracing. And so, I think we frequently have to internalize those patients pretty early on in their labor course, which I always feel hesitant about, just increasing their risk of infection, and doing that so early on. But I also understand that it can be a burden to the nursing staff, constantly trying to adjust the monitors, in order to effectively trace their contractions; and again, the heart tracing.

The primary suggestion to improve monitoring was to improve the associated equipment, such as longer belts and monitors that adapt for a patient’s size.

### Moving and positioning

Providers described various challenges in moving, positioning (and re-positioning), and transferring patients. They had trouble optimizing patients’ positioning for delivery, especially when the beds are too small and changing positions were physically difficult for the patient and the assisting provider. Providers reported asking for additional assistance because patients are numb from the epidural earlier and longer. As a result, nurses reported making fewer position changes, which concerned them because of the potential for slower cervical dilation, higher infection risk, and decreased chance of vaginal delivery. One nurse described these challenges:

I think in less emergent scenarios that also just movement, in general, doesn’t happen as frequently with our patients with high BMI’s. Movement and labor is crucial to get them to dilate, to help the baby have good heart rate. And both because I think, in general, it’s a lot of work to continually change the positions in our patients with BMIs.

Providers reported that difficult position changes could interrupt fetal monitoring and IV access, as well as negatively affect the ability to intubate patients if needed. Providers were concerned that the physical exertion needed to change position may also cause patients to lose stamina for labor.

To address these challenges, providers expressed a desire for training and guidance on the best positions. They also recommended designating additional staff to help with position changes, so the maneuvers are less physically exhausting for patients and providers alike.

### Communications

There were challenges in communication between patients and providers. On the patient side, providers felt they did not adequately counsel regarding the risks of obesity and approaches to prevent negative outcomes. Providers also acknowledged that counseling on risks was challenging with all patients, primarily because most providers do not want to focus on what could go wrong, and providers do not want to unnecessarily scare patients. Many providers felt uncomfortable mentioning weight and shied away from doing so during counseling because they didn’t want to insult patients, perpetuate fat-shaming, medicalize childbirth, or make the patient feel like there is something wrong with them. As one resident described, “I definitely have a bias towards not mentioning weight, because I feel sometimes, I don’t want to make the patient feel uncomfortable, or I don’t want to instill distrust, or have them think that I’m thinking about their weight in a negative way.” Because of this hesitance to mention weight, providers said they sometimes do not give explanations for common issues that arise in labor or obtain a comprehensive medical history.

Providers also wished to receive more feedback from patients. Reviewing patient satisfaction surveys might inform them about how patients feel about their labor experience. Some lamented not knowing how to make patients more comfortable, how to reassure them, or how patients prefer to approach such a sensitive topic in counseling. Several wondered whether avoiding weight discussions make patients feel worse or keeps the subject taboo.

Some providers felt a disconnect between themselves and their patients, mentioning how difficulties in care can affect the patient-provider relationship. Communication gaps also contributed to certain misunderstandings. As one member of the anesthesia team explained, “sometimes I wish it was easier for patients to understand anesthesiologists are there for their safety, and we’re not there to be bad guys. I think there are a lot of mommy blogs that are against epidurals and that kind of thing, so it would be helpful if they understood more often it’s for safety purposes.”

Issues in provider communication seemed to stem from lack of specific guidelines. Without guidance, providers attempted to use procedures and standards for the general population for patients with a BMI ≥ 50 kg/m^2^, like those for oxytocin titration. Many mentioned that standard counseling language for patients with a BMI ≥ 50 kg/m^2^ does not exist. Others worried the frustrations they felt from care challenges affected how they speak about patients.

To address communication issues with patients, providers wanted standardized language templates to counsel on topics like labor differences, expectations for being at the hospital (including early epidurals), monitoring, preventative actions, and outcomes. Some desired consistent, patient-centered conversations that frame high BMI as simply part of health history and normalize it. A resident explained:

And so, if maybe have training on the best way to approach those conversations, and do it in a way that is patient-centered, and is said in a way that is looking out for them, and shows that we actually–by saying these things, we care more about them, as opposed to saying it–and maybe people, I’m sure, in the past, because of their weight, have felt–just have poor experiences in healthcare because of all these things. And to have our message being received in the best way possible.

Regarding communication between providers, several explained that a quick meeting of team members or "time out" at shift change or other key times would sync the clinical concerns across all provider groups. Some wanted better interdisciplinary relationships and more opportunity for conversation among different provider groups. One OBGYN faculty member suggested the bedside nurse initiate any relevant pathway and take the lead on communications because that person “is the one running the show for the patient.”

### Suggestions for improvement

Throughout discussions of the challenges they face, providers also offered suggestions on how to improve conditions, processes, and care for patients with a BMI ≥ 50 kg/m^2^. To capture all possible suggestions, they were also asked to imagine what they would include in a pathway or toolkit for care of patients with a BMI ≥ 50 kg/m^2^. A wealth of ideas emerged, primarily pertaining to guidelines, training, checklists, and patient tools.

Providers suggested implementing guidelines or protocols for many aspects of care so that, as one OBGYN faculty member stated, “everybody can be on the same page because not all of us are consistently experienced with this” They wanted guidelines for counseling, positioning, titration of oxytocin, internal monitoring, and staffing. In the study survey, half of providers (14, 51.9%) responded “Yes” when asked if they use guidelines for care of patients with a BMI ≥ 50 kg/m^2^, but only two named specific guidelines (American College of Obstetricians and Gynecologists, Society for Obstetric Anesthesia and Perinatology). Others only mentioned small-scale guidance, like using a transfer mat or ordering an x-ray after cesarean deliveries. These varied responses further stress the need for guidelines specific to patients with a BMI ≥ 50 kg/m^2^.

An array of training was suggested. Nurses desired training on managing oxytocin titration, including information on oxytocin receptors and IV oxytocin. They also wanted training on using ultrasound to place IVs to improve the IV placement experience. Some wanted training on position changes while others wanted training on how and where to use blood pressure cuffs and how to choose proper cuff size. Providers across groups wanted training on patient-centered counseling, sensitivity training, and implicit bias training to improve their communication and relationships. One participant in the CNM/NP group explained, “Including this in implicit bias I think would be helpful. We have implicit bias training. Sizeism is definitely something that exists in our society.”

To deliver training, providers suggested creating an online module they could complete on their own time. Some suggested including video explanations from physicians to engage providers. As one nurse described:

I think nurses want to know the rationale a lot of times. I think we probably know our Pitocin policy backwards and forwards. We all know how often you can go up. We all know when you turn it down. We all know that policy. But I think sometimes, maybe especially for new nurses who are learning to understand the rationale like why do we do it this way. There is a reason for most things. Some things I guess you could question. But for most things, there are reasons why we do it. And I think having those discussions with having some of our physician partners say this is done because there were studies done and they showed blah, blah.

Providers recommended various tools and referrals. They discussed ideas for handouts such as how to prepare for induction. One participant in the CNM/NP described what a handout to prepare for induction, for example, could look like:

Ideally, there would be a handout or something that they could give to the patients. We kind of talked about this a little bit. Anyone who is being induced for whatever reason, patients don’t know a lot about inductions, in general. And for each specific category whether you’re getting induced for obesity or hypertension, it would be nice to have a handout that you could give that they could read before they come into the hospital like this is the gist, the basics. These are the issues that could be a problem during labor and what you’re at higher risk for.

One participant in the CNM/NP group stated that whatever information they provided to patients in handouts should also be available online. Providers felt creating checklists would help patients as well as themselves. One resident posited:

Like having a checklist of things that we should be even more careful about thinking of, that we articulate out loud before the case, so everything we do is intentional, related to that patient, and their risk factors, because we are intentional; but sometimes, when you have to voice it out loud, that increases the intentionality; and also, discussion in the room, if needed, to make sure that we’re making the best choice for that patient.

An OBGYN faculty member added, “Honestly, having less to think about, like having these kits or these checklists that kind of free our brain power to do other stuff, is probably better.” Table 3 shows a full list of patient tools, resources and referrals suggested by providers across groups.

**Table 3:** Suggested items for a toolkit or checklist for peripartum management of patients with a BMI ≥ 50 kg/m^*2*^.

Items	
Prior to admission	Patient counseling with handout or website access regarding risks during peripartum care and suggested adaptations
During labor and delivery	Designate room location (e.g., larger bed, easy access to OR, room with BMI items from Table 2)
	BMI cart or BMI room equipment: transfer mat, larger blood pressure cuff, larger gown, longer belts for fetal heart rate and contraction monitoring (See Table 2)
	Define nursing staff ratios (i.e., one to one nursing care during part or all of labor and recovery)
	Place mechanical VTE prophylaxis with SCDs
	Define frequency of patient position changes (i.e., every 30 or 60 minutes)
	Define monitoring plan for fetal heart rate and contractions with patient communication (e.g., when to place internal monitors, is intermittent monitoring an option?)
	Define plan for titrating oxytocin
After delivery	Incentive spirometer at bedside with education on use
	Chemical VTE prophylaxis
Continuous	Enhance communication among providers (e.g., touch point with all providers at admission, change of shift, or change in phase of care)

BMI body mass index

VTE venous thromboembolism

SCD sequential compression devices

OR operating room

## Discussion

We aimed to explore the experiences of a diverse group of providers who provide peripartum care for people with a BMI ≥ 50 kg/m^2^. We determined six key themes of staffing, equipment, oxytocin use, monitoring of vital signs, movement and positioning, and communication. Issues in one area of care often affected others and cause additional issues, compounding the risk, safety concerns, and impact on patient experience.

Providers faced two particularly troubling paradoxes. They tried to promote vaginal delivery as much as possible. However, the numerous barriers providers faced during monitoring of vital signs (e.g., blood pressures, fetal heart rate, contraction pattern) made the labor process more challenging. Similarly, epidurals are recommended early in the labor process because placement can be technically challenging, and anesthesia is then readily available in the event of an emergency [20]. However, early placement limits a patient’s mobility for a longer duration during the labor, thereby requiring more interventions on behalf of providers and potentially increasing risks for protracted labor and complications such as thromboembolism. Although several provider groups felt their actions had the safety of their patient and their fetus as a priority, they felt as though the patient perception was sometimes the opposite.

Our findings are similar to prior studies of midwives, who also identified safety issues and different equipment requirements (i.e., larger beds and chairs) as well as the need for increased skills in providing care [13, 21–23]. Prior studies have also identified themes such as a “creeping normality”, “feeling in the dark”, and referring to the obesity epidemic as a “runaway train” to describe how common obesity in pregnancy has become, the lack of guidance for management, and how rapidly the changes in weight have occurred [13]. These themes were similar to our providers whose common emotions ranged from frustration, fear, and uncertainty of how to provide a safe delivery in a stressful health environment.

The lack of existing guidelines or standardized aspects of care permeated throughout the discussions, especially for oxytocin which is typically administered through an IV and managed by nurses. Whether used for an induction or augmentation of labor, the dosing does not account for any anthropomorphic data such as a person’s weight or BMI, but instead is dosed according to a clinical effect (i.e., occurrence of contractions). Prior studies describe longer labors, slower progress in labor, and requiring more oxytocin to achieve a vaginal delivery as BMI increases [8, 24, 25]. The relationship between labor and oxytocin also involves oxytocin receptors in the myometrium and other feedback pathways including the autonomic nervous system [26]. Data to support alternative dosing regimens for oxytocin are limited. For example, a secondary analysis of a double-blinded randomized controlled trial of singletons ≥ 36 weeks of gestation evaluated high (initial and incremental rate of 6mIU/min) and standard oxytocin dose (initial and incremental rate of 2mIU/min) to determine if there was an effect modification among people with a BMI < 30 kg/m^2^ or ≥ 30 kg/m^2^ [27]. Although high dose oxytocin reduced the frequency of chorioamnionitis for people with a BMI < 30 kg/m^2^, there were no treatment effects for the outcomes of cesarean delivery, endometritis, postpartum hemorrhage, or a severe morbidity composite for people with a BMI ≥ 30 kg/m^2^ [27]. Providers who lamented the lack of guidance on oxytocin use in our study were those most involved in its dosing and titration (nurses, certified nurse midwives, nurse practitioners, resident physicians). Certainly, this topic is an area in need of further research so that oxytocin dosing is safe and effective.

The topic of communication not only related to providers, but communications between providers and patients. Discussions about weight are sensitive ones, as has been previously described in studies about appropriate terms to use for weight and how to start the discussion about weight [28, 29]. Providers felt as though no matter what words they used, they weren’t well-received and opted to avoid the topic altogether. Providers suggested tools for patient education (i.e., handouts, information on hospital website) and their own education (i.e., online modules, bias training).

Important information was gathered pertaining to how to improve care in the six thematic areas. It is possible that the feasible and tangible items for a “BMI cart” (Table 2) could be adapted for most labor and delivery sites. Items for either a toolkit or checklist (Table 3) are also thought to be practical, yet topics such as oxytocin titration require more research and nursing ratio adjustments also need to address any systems and/or financial issues.

We recognize the following limitations to this study. Patients were implicitly and explicitly missing from this work—implicitly in that the objective is to improve care for them, which was only examined from the perspective of their providers and explicitly in that multiple provider groups wondered what patients think and wanted more feedback from patients. Future studies could examine the peripartum experience of patients and their suggestions, which could inform and potentially enhance many of the improvements that providers suggested. The providers who participated in these focus groups work at a single urban hospital with a large volume of deliveries and diversity in complexity of deliveries as well as extremes of weight. As such, providers at different sites may not find these experiences similar to theirs or find the suggestions applicable to their care.

## Conclusion

In summary, this group of providers, representing diverse roles in peripartum care, discussed a range of challenges and barriers to optimal care. They also described how current approaches to care may negatively affect the peripartum experience and pose threats to safety for patients with a BMI ≥ 50 kg/m^2^ and their providers. A wealth of information was gathered regarding how to improve care in several areas.

## Supporting information

S1 File COREQ checklist. (PDF)

S2 File Focus group guide. (DOCX)

S3 File Data. (DOCX)

S4 File Codebook for focus group transcript analysis. (DOCX)
