# Supplementary material for: A qualitative exploration of the challenges providers experience during peripartum management of patients with a body mass index ≥ 50 kg/m2 and recommendations for improvement
Source: PLoS One. 2024 May 16;19(5):e0303497. doi: 10.1371/journal.pone.0303497 (PMC11098326; doi:10.1371/journal.pone.0303497)
Supplement: S2 File — (DOCX) [file pone.0303497.s002.docx]

S2 Appendix: Focus group guide

I wanted to first thank you for agreeing to participate in this focus group today. Before we get started, I wanted to give you a little bit of information about what to expect from the discussion.

We are interested in hearing your experiences providing care to pregnant patients with a BMI over 50 and your feedback on optimizing their care.

There are no right or wrong answers. We want to know about your experience and what you think.

I will audio record this discussion to ensure that your input is well documented. In any reports of this research (including any transcription or summary of the discussion), your responses will not be linked with your name or any personally identifying information.

If you could, please say your name before sharing your comments the first few times around to help to differentiate between participants on the recording.

Please take turns when talking and try not to talk over each other since that will also make it difficult when we revisit the recording.

Finally, I want to remind everyone to respect each other’s privacy. What’s discussed in the group today should stay amongst those of us in the room and shouldn’t be discussed outside of the focus group. Also, just a reminder to please be respectful of the others during the discussion.

Do you have any questions before I start the recording?

1. Pregnant women with super morbid obesity can be challenging to care for during labor and delivery for many reasons, and sometimes this may lead to complications. I would first like to hear about some of the most challenging situations you have faced in the clinical care of women with extreme (SMO) obesity during delivery?

During a cesarean delivery?

After delivery?

Probes: What were the outcomes? What could have been done to better prepare for what happened? (e.g. ordering more tests or different tests or consulting other providers)

2. Some of the approaches we currently practice in labor/delivery for women with extreme obesity include use of special equipment such as “Hover mats” and placing a note in the EPIC L&D greaseboard with “BMI”.  Which of these approaches do you think is helpful vs. not helpful for clinical care?

What about other approaches? *If others listed:* What is helpful or not helpful for clinical care?

Probes: What else should we be doing to improve peripartum experience of (a) patients and staff and (b) clinical outcomes? Can you think of any other strategies?

3. What do you think patients and their families know about the risks during labor and delivery and postpartum for women with extreme obesity?

Probes: What do you wish patients and their families knew about their risks? How do you counsel patients about these risks? How does the patient’s family or social support system influence your care? How can patient preparation or counseling for risks affect your clinical care or outcomes?

4. Strategies that have been shown to reduce adverse outcomes in obstetrics include creating checklists or toolkits. After identifying patient, provider, and systems factors in the care pathway, the recommendations are implemented into care. For example, we at NMH have designed this type of pathway for shoulder dystocia and management of hypertension. If we could design a toolkit or maternal care pathway to improve clinical care of women with extreme obesity at NMH and nationwide during their peripartum course, what would it look like?

Probes: What would you include in it? Who needs to be aware of/use the toolkit? How would it work? Do you think it will work? Why or why not?

5. Is there anything else that anyone would like to share with us about anything we have discussed here today?

Thank you again for speaking with us. We appreciate your time and perspective.
