# Supplementary material for: A qualitative exploration of the challenges providers experience during peripartum management of patients with a body mass index ≥ 50 kg/m2 and recommendations for improvement
Source: PLoS One. 2024 May 16;19(5):e0303497. doi: 10.1371/journal.pone.0303497 (PMC11098326; doi:10.1371/journal.pone.0303497)
Supplement: S3 File — (DOCX) [file pone.0303497.s003.docx]

| Record ID | Provider Type | Which best describes your role in providing care at NMH: | What is your ethnicity? | Race: | Gender: | What is your age? | How many years have you been practicing at NMH? | When (what year) did you complete your training? | Do you make any modifications for the intrapartum care of women with a BMI ≥ 50 kg/m^2? | Do you make any modifications for the postpartum care of women with a BMI ≥ 50 kg/m^2? | If yes to #1 or #2, what are the modifications? | In your practice, do you request transfer of care to another provider or specialty if the women's BMI is too high? | If yes to #3, what is the BMI cut-off that you use to transfer care? | If yes to #3, what providers do you transfer to? | If yes to #3, what is the reason for transfer of care? | Are there any specific guidelines that you follow for the peripartum care of women with a BMI ≥ 50 kg/m^2? | If yes to #4, please list or describe your resources. |
| --- | --- | --- | --- | --- | --- | --- | --- | --- | --- | --- | --- | --- | --- | --- | --- | --- | --- |
| 1 | Nurse/Surgical Assistant | RN | Non-Hispanic | White | Female | 43 | 17 | 2005 | Yes | Yes | Utilize alternate equipment, medications when necessary. For example, alternate gowns, labor monitoring equipment, additional staff for procedures/moving patients when needed. | No |  |  |  | No |  |
| 3 | Nurse/Surgical Assistant | RN | Non-Hispanic | White | Female | 26 | 3.5 | 2019 | Yes | Yes | A hovermat is applied to the patient bed for anyone with a BMI>40, a postop X-RAY is performed for c-section patients, and occasionally the provider is contacted to discuss internal monitors if monitoring the baby externally is interfering with or limiting position changes the patient is doing. | Not Applicable |  |  |  | Not Applicable |  |
| 5 | Nurse/Surgical Assistant | RN | Non-Hispanic | White | Female | 27 | 2 | 2020 | Yes | No | using two sets of bands to avoid pressure injuries on the skin. using BMI gowns, two IVs. Still encouraging movement despite BMI | Not Applicable |  |  |  | Yes | Hovermat |
| 6 | Nurse/Surgical Assistant | RN | Non-Hispanic | white | female | 63 | 40 | 1981 | Yes | Yes | 2 ivs  hoover mat  different size B/P cuffs  x-large gowns | Not Applicable |  |  |  | Yes | type and screen  more of a prep for a C/S  xray after a C/S to be sure no laps or insturments or needles got left inside |
| 7 | Nurse/Surgical Assistant | RN |  |  |  | 28 | 3.5 | 2019 | Yes | Yes | Intrapartum, I have to get creative with monitoring and getting epiduralized patients into different positions while still being able to monitor the fetus. We often have to use multiple sets of belly bands to secure the EFM to the patient and I wish there were a better, more comfortable way to do this. The bands always dig into their backs. | Not Applicable |  |  |  | Yes | Use of a hovermat for laboring/IOL patients or scheduled c sections |
| 8 | Nurse/Surgical Assistant | RN | Non-Hispanic | White | Female | 24 | 2.5 | 2020 | No | No |  | No |  |  |  | Yes | Using a hovermat is BMI > 40, additional skin prep time for c sections, increased antibiotic dose |
| 9 | Anesthesiology | Fellow - Obstetrical Anesthesia | Non-Hispanic | White | Female | 35 | 3 | 2020 | Yes | No | Recommend early epidural  Second peripheral IV  SDB screen  Hovermat  Possibly decreased intrathecal medication dosing | No |  |  |  | Yes | As above |
| 10 | Anesthesiology | Faculty - Obstetrical Anesthesia | Non-Hispanic | Caucasian | Female | 52 | 16 | 2007 | Yes | Yes | Preanesthesia consultation, second peripheral IV, hover mat, encourage epidural placement, appropriate postpartum unit (OBIMU v PP), VTE prophylaxis | Not Applicable |  |  |  | Yes | ACOG guidelines |
| 11 | Anesthesiology | Faculty - Obstetrical Anesthesia | Non-Hispanic | White | Male | 45 | 6 | 2016 | Yes | Yes | In general: obtain additional IV access, examine airway more frequently intrapartum, have low threshold to transfer to IMU for continuous pulse oximetry after delivery. | Not Applicable |  |  |  | No |  |
| 12 | Anesthesiology | Fellow - Obstetrical Anesthesia | Non-Hispanic | Caucasian | Female | 31 | 0.75 | 2022 | Yes | Yes | Advocate for early epidurals due to potential technical difficulty, assess airway exams on the labor floor more frequently, potentially place additional IV access if access is deemed difficult, consider VTE prophylaxis, closely monitor sedating medications specifically in those with known sleep apnea. Will consider down-dosing intrathecal spinal doses in the operating room, with placement of epidural catheter in the event that anesthetic level needs to be increased. | No |  |  |  | Yes | -pulse ox monitoring with intrathecal opioids  -VTE prophylaxis |
| 13 | Anesthesiology | Fellow - Obstetrical Anesthesia | Non-Hispanic | White | Male | 31 | 6 | 2021 | Yes | Yes | More frequent rounding on epidural function Assessment of SDB risk, consideration of continuous pulse ox if duramorph is administered | No |  |  |  | Yes | SOAP guidelines for obese parturients |
| 14 | Physicians | Faculty - OB/GYN | Hispanic | White | Female | 50 | 17 | 2006 | Yes | Yes | anesthesia team and the ob and the nursing team ensure all precautions are taken, accomodations (hovermat etc.) to transfer patient , epidural dosage, appropriate OR table extensions and tools such as the suspenders to lift the panis if needed in an emergent c-section scenario etc.  dvt ppx post partum, other accomodations as needed | Not Applicable |  |  |  | No |  |
| 15 | Anesthesiology | Fellow - Obstetrical Anesthesia | Hispanic | White | Female | 31 | 5 | 2022 | Yes | Yes | Intrapartum we encourage early epidural placement to avoid any airway manipulation that would accompany a general anesthetic during a crash c section. We also ensure 2 large bore IVs. Patients with BMI over 50 are routinely seen as part of an anesthesia consult prior to delivery to discuss these modifications. Post partum, especially after c sections they are monitored more closely for respiratory depression | No |  |  |  | Yes | There are recommendations for post partum monitoring if duramorph is administered for c section |
| 17 | Midlevel | CNM or NP | Non-Hispanic | Human | Female | 45 | 20 | 2008 | Yes | Yes | less position changes IP due to FHT, internal monitors for more accurate FHT. | No |  |  |  | No |  |
| 18 | Midlevel | CNM or NP | Non-Hispanic | White | Female | 47 | 3 | 2003 | Yes | Yes | VTE Protocol | Not Applicable |  |  |  | Not Applicable |  |
| 19 | Midlevel | CNM or NP | Non-Hispanic | White | Female | 36 | 8 | 2015 |  | Yes | Hover mat for bed, Lovenox PP for VTE prophylaxis. | No |  |  |  | Yes | Weekly NST starting at 37 weeks  Nutrition counseling  Induction 39-40 weeks |
| 20 | Residents/Fellows | Resident physician - OB/GYN | Non-Hispanic | Black | Woman | 27 | <1 | 2022 | Yes | No | Allow more time before calling arrest because want to give more time on pitocin. | No |  |  |  | No |  |
| 23 | Midlevel | CNM or NP | Non-Hispanic | Caucasian | Female | 48 | 10 | 2000 | Yes | Yes | Hover mat and lovenox for DVT prophylaxis. | No |  |  |  | No |  |
| 24 | Midlevel | CNM or NP | Non-Hispanic | White | Female | 38 | 8 | 2021 | Yes | Yes | Patients placed on hover mats, pre anesthesia consults. | No |  |  |  | No |  |
| 26 | Midlevel | CNM or NP |  |  | female | 55 | 24 | 1992 | Yes | Yes | Appropriate sized BP cuff; IUPC if indicated; Hovermat | Not Applicable |  |  |  | Not Applicable |  |
| 27 | Midlevel | CNM or NP | Non-Hispanic | Caucasian/  white | Female | 47+ | 8+ | 2011 | Yes | No | Only have NSVD's.  -Hover mat ≥40  -'BMI'  - grease board flagged | Yes | 40 prepregnancy | OB physician | BMI | Yes | I think there is a policy? |
| 28 | Residents/Fellows | Resident physician - OB/GYN | Non-Hispanic | Caucasian | Female | 28 | 1 | 2022 | Yes | No | Intrapartum ensure patient has hovermat, modify pushing positions to accommodate habitus | No |  |  |  | Yes | Hovermat intrapartum. If s/p c/s, closing xray. |
| 29 | Residents/Fellows | Resident physician - OB/GYN | Non-Hispanic | Black | Female | 29 | 4 | 2019 | Yes | Yes | Hovermat  VTE prophylaxis  Encourage early epidural  Lower threshold to place IUPC/FSE  Abdominal tape at time of c/s | No |  |  |  | Yes | Hovermat |
| 30 | Physicians | Faculty - OB/GYN | Non-Hispanic | White | Female | 40 | 7 | 2014 | Yes | No | 1) Routine use of internal monitors (IFE and IUPC) and expectation of higher dose of oxytocin required | No |  |  |  | Yes | Antenatal surveillance with NSTs for BMI >40, Routine growth US q trimester |
| 31 | Physicians | Faculty - OB/GYN | Non-Hispanic | White | Male | 52 | 17 | 2005 | Yes | Yes | Additional monitoring antepartum, modify surgical approach if c/s, VTE | No |  |  |  | No |  |
| 32 | Physicians | Faculty - OB/GYN | Non-Hispanic | white | female | 32 | 2 | 2021 | Yes | Yes | VTE prophylaxis - SCDs in labor if anticipating long course, lovenox pp, oral antibiotic prophylaxis pp if c/s | No |  |  |  | No |  |
| 33 | Residents/Fellows | Resident physician - OB/GYN | Hispanic | White | Male | 28 | 1 | 2022 | Yes | Yes | -Increased position changes  -Teaching on decreasing infection risk | Not Applicable |  |  |  | No |  |
