# Supplementary material for: A qualitative exploration of the challenges providers experience during peripartum management of patients with a body mass index ≥ 50 kg/m2 and recommendations for improvement
Source: PLoS One. 2024 May 16;19(5):e0303497. doi: 10.1371/journal.pone.0303497 (PMC11098326; doi:10.1371/journal.pone.0303497)
Supplement: S4 File — (DOCX) [file pone.0303497.s004.docx]

#### S4 File: Codebook for focus group transcript analysis
